# 21. A case of autoimmune encephalitis in relapsing polychondritis

**DOI:** 10.1093/rap/rky033.013

**Published:** 2018-09-20

**Authors:** Cattleya Godsave, Raj Ullegaddi, Prateek Sharma, Kevin Fairburn

**Affiliations:** 1 Rheumatology, Chesterfield Royal Hospital, Chesterfield, UNITED KINGDOM; 2 Care of the Elderly, Chesterfield Royal Hospital, Chesterfield, UNITED KINGDOM; 3 Radiology, Chesterfield Royal Hospital, Chesterfield, UNITED KINGDOM

**Introduction:** We report a case of autoimmune (limbic) encephalitis associated with relapsing polychondritis (RP) that presented as an emergency. The case illustrates the importance of a low threshold for recognising neurological complications of autoimmune disease, especially on a general medical take and raises questions about the most appropriate treatment strategy of this rare condition. A patient with RP and its associated complications may present to various specialties. The management of neurological complications resides in a multidisciplinary approach requiring good communication between general medical and specialist teams. To many general physicians and indeed rheumatologists and neurologists, neurological presentations in the context of autoimmune disease can be difficult to diagnose and manage. RP is considered to be a rare multisystem autoimmune disorder characterised by progressive inflammation and destruction of cartilaginous tissues. The main structures involved include the ears, nose, larynx and tracheobronchial tree. It can be difficult to diagnose due to the relapsing nature of the condition often with a prodromal phase of non-specific symptoms including joint pain, fever and anorexia. Neurological involvement in RP is rare and variable in presentation. There is limited literature upon which to base clinical practice and we present our experience in this case.

**Case description:** Our patient, a 70 year old man, initially presented to the ear, nose and throat (ENT) team with an episode of fever, bilateral swollen ears and otitis media. He was discharged that day with oral antibiotics. He had recently experienced two brief episodes of visual loss in the left eye treated as embolic phenomena. He presented to hospital one month later as a potential stroke and the on call medical team assessed his case for thrombolysis. His complaints on this admission were headache, confusion, slurred speech and right sided paraesthesia on a background of general decline for the preceding few weeks. He scored one on the National Institute of Health Stroke Scale (NIHSS) to assess for stroke symptoms. He displayed cerebellar signs of dysdiadokinesis, inability to perform heel-shin movements and ataxia. He also demonstrated the classical ear deformities associated with relapsing polychondritis. At this stage it also became apparent that he had suffered a three year prodromal illness with recurrent lower respiratory tract infection, bronchiectasis, sinusitis, anaemia, blepharitis and remitting polyarthritis. Blood tests showed a normocytic anaemia Hb 101g/L (130-180), WBC 8.7 x 10^9^/L (4-10), platelets 550 x 10^9^/L (150-400), ESR 99 mm/hr (1-10), and CRP 36 mg/L (<10). Renal, liver, thyroid function, haematinics and immunoglobulins were all normal. ANA, rheumatoid factor and ANCA tests were negative. CT head scan showed a subtle area of loss of grey-white matter differentiation in the left occipital region, which may have indicated a hyperacute infarct. He was treated with aspirin 300 mg daily and underwent an MR scan of his brain which showed some minor ischaemic changes and mild atrophy of the right hippocampus. The scan also demonstrated appearances of auricular inflammation, consistent with RP. A lumbar puncture was performed and cerebrospinal fluid (CSF) showed protein 0.57g/L (0.15-0.45) and WBC 55/cm^3^, predominantly lymphocytes. Viral serology and microbiology performed on the CSF sample was negative. A CT chest-abdomen-pelvis showed a probable inflammatory abnormality in the upper lobe of the right lung, an old traumatic crush fracture of the L3 body and no evidence of malignancy. He was treated with antibiotics for a potential exacerbation of bronchiectasis in view of the CT findings. Serum neuronal autoantibodies including GAD, voltage gated potassium channel, Purkinji cell, anti-Yo, neuronal nuclei, anti-Hu/Ri, amphyphisin, anti-CV2/CRMP-5, anti-PNMA2 (Ma2/Ta) and anti-Tr were subsequently returned negative. A diagnosis of autoimmune encephalitis associated with relapsing polychondritis was made. He was treated with intravenous methylprednisolone 1 g daily for three days followed by oral prednisolone 60 mg daily and discharged following a significant improvement in his neurological status. On review in out patients after a month of oral steroids his neurology had improved such that he enjoyed walking for up to seven hours twice a week. His Addenbrooke’s cognition score improved from 82 to 92. He was left with some residual signs of dysdiadochokinesis in the left arm and unsteadiness on heel-toe walking. The dose of prednisolone was reduced to 40 mg daily and he was given intravenous zolendronate 5 mg rather than oral bisphosphonate therapy due to known Barrett’s oesophagus. After three months he presented with a significant relapse of his condition having taken prednisolone 30 mg daily. He reported reduced mobility, and showed signs of marked ataxia, such that he was virtually bed bound, as well as more noticeable cognitive impairment. Blood tests showed Hb 121g/L (130-180), platelets 366 x 10^9^/L (150-400), ESR 57 mm/hr (<10), compared to ESR 23 mm/hr a month previously. An MR scan of brain with contrast showed a slight increase in white matter T2 signal abnormality when compared to his previous MR scan. The appearances were considered non-specific. Lumbar puncture again showed no sign of infection. He was re-treated with three doses of methylprednisolone 1 g daily and in addition intravenous immunoglobulin (IVIg) 2 mg/kg over five days. He was discharged following clinical improvement on prednisolone 60 mg daily and received another course of IVIg after six weeks. Mycophenolate mofetil 1 g twice daily was added to a reducing dose of prednisolone. We avoided methotrexate and other cytotoxic therapy in view of his history of bronchiectasis. The patient continued to make a gradual improvement managing a five mile walk on rough ground with a walking aid and experiencing only mild cognitive impairment nine months after his initial neurological presentation.

**Discussion:** This case highlights the difficulty of diagnosing and treating severe and rare neurological complications of autoimmune disease adding a level of complexity to the usual conundrum of diagnosing RP, a rare condition in its own right. Autoimmune encephalitis is a condition most rheumatologists and general physicians are unlikely to be very familiar with. Our patient initially presented to the ENT team with bilateral auricular swelling. In retrospect we believe he had a prodromal illness for about three years with systemic features, upper and lower respiratory tract and musculoskeletal involvement. The case emphasises the varied presentation of a rare condition to numerous specialties. As part of the general medical workforce it is essential to be aware of potential cerebral manifestations of autoimmune disease and have a low threshold for suspecting these in a patient with a concurrent autoimmune condition, especially in an evolving phase of their illness. The neurological manifestations of RP are interesting in themselves as the pathogenesis is not fully understood. Treatments for autoimmune encephalitis are mainly empirical, due to the scarcity of cases and literature. Often high dose pulse steroids are the mainstay of treatment, although IVIg and other immunosuppressive and cytotoxic agents have been used.

**Key Learning Points:** RP is a rare but extremely diverse condition with potentially fatal complications. General physicians and specialists should have a low threshold for suspecting neurological involvement in an autoimmune disorder and investigate thoroughly when appropriate. Although pulse doses of methylprednisolone and high dose oral prednisolone are a recognised treatment for autoimmune encephalitis, our patient relapsed soon after an initial very good response. Based on our experience in this case, and in similar circumstances, we would favour adding IVIg to the steroid regimen at an early stage in treatment rather than following a relapse. The absence of specific indices of disease activity or damage in this condition adds to the complexity of its management. However, close monitoring is required to ensure the resolution of symptoms and signs of the condition and also that inflammatory markers are improving. Long term immunosuppressive therapy, such as mycophenolate mofetil used in our case, has an empirical role to play. As with other autoimmune conditions, a multidisciplinary approach should be adopted and we found this especially important in our case between general medical, rheumatology, radiology and neurology clinicians.

**Disclosure: C. Godsave:** None. **R. Ullegaddi:** None. **P. Sharma:** None. **K. Fairburn:** None.

